# Structures and Functions of the Envelope Glycoprotein in Flavivirus Infections

**DOI:** 10.3390/v9110338

**Published:** 2017-11-13

**Authors:** Xingcui Zhang, Renyong Jia, Haoyue Shen, Mingshu Wang, Zhongqiong Yin, Anchun Cheng

**Affiliations:** 1 Research Center of Avian Disease, College of Veterinary Medicine of Sichuan Agricultural University, Wenjiang District, Chengdu 611130, China; zhangxc923@163.com (X.Z.); shenhaoyue0724@163.com (H.S.); mshwang@163.com (M.W.); 2 Institute of Preventive Veterinary Medicine, Sichuan Agricultural University, Wenjiang District, Chengdu 611130, China; 3 Key Laboratory of Animal Disease and Human Health of Sichuan Province, Wenjiang District, Chengdu 611130, China; yinzhongq@163.com

**Keywords:** flavivirus, envelope protein, envelope domains I/II/III, membrane fusion, viral infection

## Abstract

Flaviviruses are enveloped, single-stranded RNA viruses that widely infect many animal species. The envelope protein, a structural protein of flavivirus, plays an important role in host cell viral infections. It is composed of three separate structural envelope domains I, II, and III (EDI, EDII, and EDIII). EDI is a structurally central domain of the envelope protein which stabilizes the overall orientation of the protein, and the glycosylation sites in EDI are related to virus production, pH sensitivity, and neuroinvasiveness. EDII plays an important role in membrane fusion because of the immunodominance of the fusion loop epitope and the envelope dimer epitope. Additionally, EDIII is the major target of neutralization antibodies. The envelope protein is an important target for research to develop vaccine candidates and antiviral therapeutics. This review summarizes the structures and functions of ED I/II/III, and provides practical applications for the three domains, with the ultimate goal of implementing strategies to utilize the envelope protein against flavivirus infections, thus achieving better diagnostics and developing potential flavivirus therapeutics and vaccines.

## 1. Introduction

Together with the *pestivirus* and *hepacivirus*, the *flavivirus* genus is a member of the *Flaviviridae* family. To our knowledge, it is the biggest genus and is comprised of more than 70 viruses including the arthropod-borne viruses that mainly cause severe vertebrate diseases transmitted by mosquitoes and ticks. These viruses mainly cause encephalitis and haemorrhagic fever [1]. Most flaviviruses are zoonotic, meaning that infections may spread between animals and humans [2,3]. Many flaviviruses are associated with human diseases [4,5]. Presently, the yellow fever virus (YFV), Dengue virus (DENV), West Nile virus (WNV), tick-borne encephalitis virus (TBEV), Japanese encephalitis virus (JEV) [6,7,8], Tembusu virus (TMUV) [9], and Zika virus (ZIKV) [10,11] are the most important arboviruses that threaten humans and animals in certain regions of the world, causing public health burdens and veterinary concerns. Thus, there is an urgent need for drugs or therapies to combat these diseases.

## 2. *Flavivirus* Genome and Encoded Proteins

**Figure 1:**
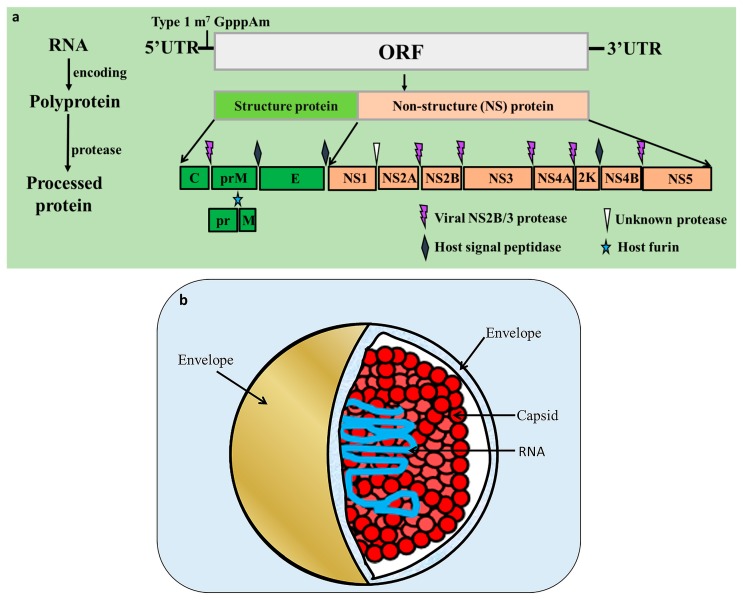
*Flavivirus* genome structure and virion. (**a**) The *flavivirus* genome consists only of an open reading frame (ORF) flanked by 5’ and 3’ untranslated regions (UTRs). The 5’UTR contains a type I cap structure (m^7^ GpppAm), and the 3’UTR lacks a polyadenylated (polyA) tail [25,26]. The polyprotein encoded by the ORF is processed into three structural proteins (C, prM, and E) and at least seven non-structural proteins (NS1, NS2A/B, NS3, NS4A/B, and NS5) by viral (NS2B-NS3) or host cellular proteases (host signal peptidase and host furin); (**b**) the C protein is responsible for coating the viral nucleic acid, and the E protein forms various symmetric structures.

Flaviviruses are enveloped, positive-sense single stranded RNA viruses with a genome of approximately 9.4–13 kb in length. The virion diameter is about 50 nm [12]. The *flavivirus* genome contains only one open reading frame (ORF) flanked by 5’ and 3’ untranslated regions (UTRs) [13], and some flaviviruses, such as JEV and WNV have −1 open reading frame shift events during translation [14]. The ORF encodes a polyprotein that is processed into three structural proteins (a nucleocapsid protein, C; a precursor membrane glycoprotein, prM; and a glycosylated envelope protein, E), as well as seven non-structural (NS) proteins (NS1, NS2A/B, NS3, NS4A, 2K, NS4B, and NS5) by viral (NS2B-NS3) or host proteases (host signal peptidase and host furin), although the protease for NS1-NS2 processing is unknown [15,16] (Figure 1a). The C protein is responsible for encapsidation to protect the genetic material (Figure 1b). PrM, which is formed by protease hydrolysation during late viral infection, participates in forming the viral envelope and plays an important role in maintaining the E protein’s spatial structure [17,18]. Both prM and E form the surface structure of virions [19]. The surface structural protein-E facilitates membrane fusion between the virus and host cell [20,21,22], and is the primary viral protein against which neutralizing antibodies are produced [23] and is indispensable in *flavivirus* biology [24]. The non-structural proteins coordinate the intracellular aspects such as viral replication, assembly, proteolysis, maturation, and host immunity regulation [18].

## 3. *Flavivirus* Envelope Glycoprotein Structure and its Role in Viral Infection

**Figure 2:**
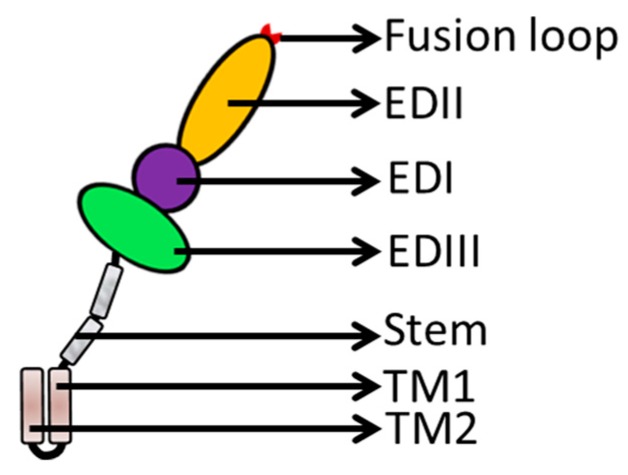
E protein structure. The E protein contains three distinct domains (EDI, purple; EDII, orange; and EDIII, green) and helix-transmembrane domains (TMDs, brown), which are linked by the stem region (grey).

The E protein forms a raft-like structure that exists as 90 anti-parallel homodimers on the viral membrane that are 170 Å in length [27,28]. The E protein is normally 53–60 kd depending on the number of glycosylation sites. Each *flavivirus* E protein monomer is organized into three structurally distinct envelope domains I, II, and III (EDI, EDII, and EDIII) (Figure 2), as determined by X-ray crystallography [29], electron cryo-microscopy [30], and NMR spectroscopy [31]. The three domains are connected by flexible hinges that mediate irreversible conformational changes during the viral life cycle [32], and all three domains are connected to the viral membrane through a helical anchor [33]. In the acidic endosomal environment, the E dimer exposes the highly conserved fusion peptide (FP) at the tip of EDII stretching from residues 98 to 112 [34].

**Figure 3:**
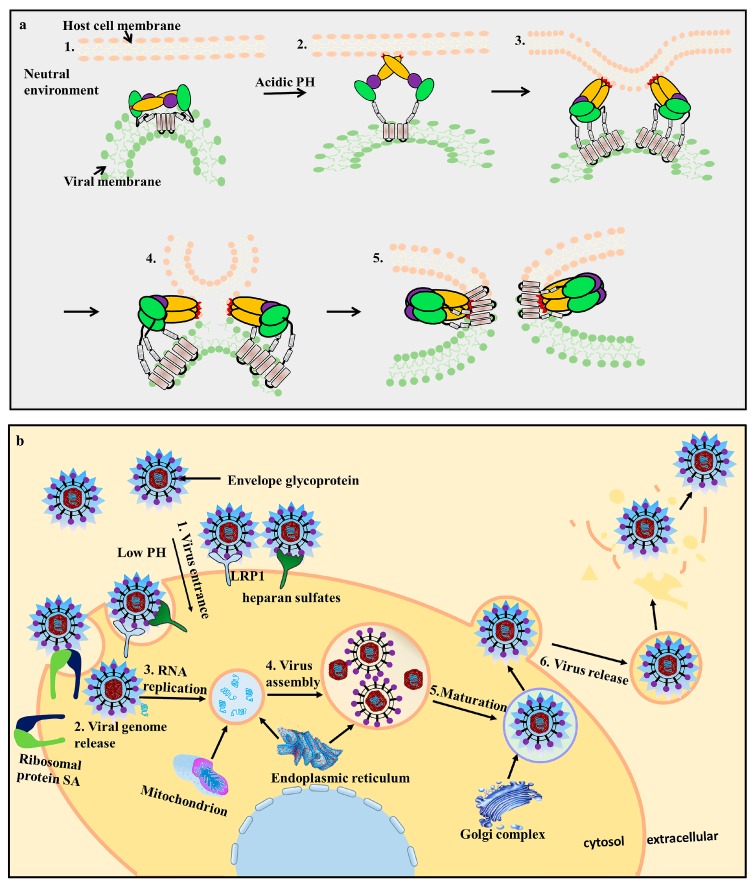
Conformational changes in the E protein during the fusion process (**a**) and *flavivirus* infection of a host cell (**b**). (**a**) The E protein undergoes conformational changes during fusion [35]. 1. In the neutral environment, the E protein monomers dimerize with each other and are anchored via the transmembrane domain; 2. The EDII fusion loop (FL, red) is exposed to the extracellular environment under low pH conditions. The E protein undergoes irreversible conformational changes and forms a hairpin-like structure, while the FL adsorbs the host cell membrane; 3. The E protein changes from a dimer to a trimer; 4. The viral and host cell membranes fuse; 5. Post-fusion formation. (**b**) viral infection of host cells is mediated by receptor-mediated endocytosis. The E protein is responsible for viral attachment, membrane fusion, and virion assembly; 1. When the virus enters the host cells, the E protein interacts with cellular receptors, such as lipoprotein receptor-related protein 1 (LRP1), heparan sulfate, and ribosomal protein SA (RPSA). Low pH conditions trigger the viral envelope to fuse with the endosomes; 2. Release of viral genome RNA; 3/4. The virus replicates and assembles by budding into the endoplasmic reticulum (ER) in an immature non-infectious formation; 5. The progeny viruses mature in the Golgi complex; 6. The progeny viruses are then transported to the cell surface for release by exocytosis.

Flavivirus E proteins belong to the class-II fusion protein, which has a unique structure with a double membrane spanning the C-terminal anchor. Following the EDI/EDII/EDIII domains is a stem region that contains two cationic amphipathic helix-transmembrane domains (TMDs, TM1, and TM2) [5]. TM1 is the stop transfer sequence, and TM2 is the internal signal sequence (Figure 2) that directs the proper processing and localization of the NS1 protein [35]. The E structural rearrangements involve a unique portion of the transmembrane segment [21,34,36], which forms a hairpin-like structure and transforms into a trimer under low pH conditions to increase particle infectivity [37]. The EDI, EDII, EDIII, and TMDs of the E protein play significant roles in membrane fusion and mediate irreversible conformational changes during the fusion process (Figure 3a). The carboxy-terminal end of the E ectodomain contains two α-helical (α1 and α2) stem regions located on the viral membrane and the transmembrane region [38]. The E protein is pivotal during viral infection (Figure 3b).

The E protein possesses four histidine residues at positions 144, 246, 284, and 319, which are located at the E dimer interface interdomain and are conserved among all *flavivirus* E proteins [39,40]. These conserved histidines may be functionally relevant to both the viral uncoating step during the early stage of the *flavivirus* lifecycle and to regulating E protein trimerization under acidic pH conditions [40,41]. Biochemical studies [42,43] have also revealed that temperature and chemicals (such as formalin or H_2_O_2_) alter the E protein structure to inactivate the viruses, suggesting the E protein’s importance during *flavivirus* infection. The multifunctional E protein has both receptor-binding and fusogenic properties [44], as well as a critical role in eliciting neutralizing antibodies [7]. The E protein is also responsible for directing viral attachment, membrane fusion [34], penetration, haemagglutination, and host range and cell tropism [23], and is associated with viral virulence, attenuation [27], virion assembly [45], stability, maturation [21], and tissue tropism [46,47].

### 3.1. EDI Stabilizes the Overall Orientation of the Protein and Related to Virus Production, pH Sensitivity, and Neuroinvasiveness

EDI is located at the N-terminus of the E protein but is situated in the middle of the E protein in the spatial configuration and forms an eight-stranded β-barrel structure to act as a bridge-like hinge. EDI contains 120 residues in three segments (residues 1–51, 137–189, and 285–302) [27] and is predominantly composed of type-specific non-neutralizing (non-NT) epitopes [48,49]. EDI is flanked on one side by the elongated dimer EDII and on the other side by the immunoglobulin-like EDIII [7,50,51]. As a central unit, EDI stabilizes the overall orientation of the E protein [39] and participates in its conformational changes [28]. EDI carries a predicted and comparatively conservative N-linked glycosylation site at residue Asn 154, consistent with most flaviviruses (DENV occurs at Asn67 and Asn153) [52]. Viruses with substitutions at these residues to amino acids that are not glycosylated display decreased levels of cellular attachment [53,54] and neurovirulence in mice [55,56]. These demonstrate that the glycosylation sites are related to virus production, pH sensitivity, and neuroinvasiveness [54]. In Leslie Goo’s study [33], a single residue lying in the EDI-EDII hinge region changes during conformational dynamics to alter the neutralization sensitivity and stability of WNV and DENV virions, and the EDI-EDII hinge is also involved in E protein movements during virus entry [57,58]. Cell surface glycosaminoglycans (GAGs) are important receptor molecules in this interaction [59,60], and this distinct sequence element may be involved in various membrane fusion and receptor binding steps. Glycosaminoglycan-binding affinity by E proteins is determined by multiple regions including the fusion gene (FG) loop of EDIII.

### 3.2. EDII Contributes to Virus-Mediated Membrane Fusion

Two elongated loops between the three EDI segments form the finger-like dimerization domain II. EDI and EDII are discontinuous peptides connected by four peptide linkers to form the EDI/EDII hinge [61]. To promote membrane fusion and virus entry, the EDI-EDII hinge region, which contains a complex quaternary epitope, undergoes complex conformational changes during the low pH-triggered late endosome process [37,62]. EDII contains an S-S bridge stabilized loop at its distal end and functions as a highly conserved internal fusion peptide (FPs) or fusion loop (FL) in amino acids 98–110 [35,63,64]. The FPs interdigitate with a hydrophobic pocket provided by EDIII-EDI [37], and this structure is involved in viral interactions with a cellular receptor and contributes to virus-mediated membrane fusion (Figure 3b). It interacts with prM, blocking the fusion loop (FL) in immature particles during cellular transportation, promoting further internalization of the virus and dictating dimer formation [18]. The hydrophobic FL is a highly conserved epitope across all flaviviruses [5]. The hydrophobic residues of FL, including W101, L107, and F108, are highly conserved among most human-infecting flaviviruses including YFV, DENV-4, and WNV [65,66]. When the virus enters the target host cell, the distal β-barrel hydrophobic FL of EDII is exposed and inserts into the host cellular membrane under certain environmental conditions. Many predominately *flavivirus* cross-reactive peptides exist in the EDII domain and stimulate the neutralizing antibodies [49]. EDII is also responsible for anti-parallel E protein homodimerization, and mutations will impact viral replication and reduce virulence [39].

### 3.3. EDIII Participates in Receptor Recognition and is Used as an Antigen

Globular EDIII is connected by a flexible structure to the opposite side of the EDI domain and is located at the C-terminus of the E protein. EDIII contains approximately 100 amino acids [67]. EDIII is anchored at the C terminus to the two “stem“ helices and two transmembrane helices [68] (Figure 2) and is stabilized by disulfide bridges. EDIII has a β-barrel shape formed by six anti-parallel β-strands (β1, β2, β3, β4, β5, and β6) [54]. The β-strands are closed to the N-terminal residues and fold into an immunoglobulin-like conservative and relatively independent domain which is thought to interact with cellular receptors [47,69]. EDIII vertically stretches out of the smooth particle surface to form apophysises, which include the type and subtype epitopes that induce specific neutralizing antibodies.

EDIII also contains important linear antigenic epitopes that directly interact with potent neutralizing antibodies [70]. These epitopes are the main target cell receptor-binding sites that assist viral entry into host cells [71]; the target cell surface receptors include heparan sulfates, ribosomal protein SA, carbohydrate receptors, and low-density lipoprotein receptor-related protein 1 (LRP1) [63]. Some scientists [72] develop peptides or monoclonal antibodies reacting against EDIII. EXE/DPPFG is a cross-reactive and immunodominant epitope that is highly conserved among flaviviruses [24] and has been confirmed by dot-blot assays in various flaviviruses using duck Tembusu-positive serum that reacts with the epitope [73]. Because of this, EDIII is used as an antigen for serologic diagnosis and is a potential candidate for a preventative *flavivirus* vaccine [23]. A previous DENV study [74] proved that most DENV-neutralizing Abs targeted EDIII; however, these findings were inconsistent with another study [75] that found that neutralizing Abs also interact with EDII, indicating that other regions of the E protein may participate in the immunoreaction. Research on the YFV 17D vaccine strain found that EDIII enhances viral binding to GAGs to the cell surface, attenuating virulence and impeding viral dissemination [51].

Mutations in EDIII affect host cell tropism and virulence, which has been reported in YFV and DENV, allowing the virus to escape antibody neutralization, which has also been reported for JEV, TBEV, and DENV, and these data showed that EDIII is invaluable in the viral lifecycle. Interestingly, based on the EDIII domain that inhibits the infectivity of cognate viruses, such as DENV, WNV, YFV, and JEV, some researchers have proposed that EDIII could potentially be used as a therapeutic molecule in antiviral research. More studies on *flavivirus* EDIII have been executed with YFV [31], DENV [76,77], WNV [78], and JEV [43], and have revealed few differences in EIII functions. The neutralizing epitope region is particularly conserved across viruses. For instance, the neutralizing epitopes in EDIII contain the residues 306, 307, 308, 330, 332, 366, 391 of WNV [79]; 306, 331, 333, 337, 360, 373–399, and 387 in JEV [27]; and residues 307, 333–351, and 383–389 in DENV [80]. To reduce the risk of cross-reactive antibodies, some researches have paid attention to EDIII [77]. In previous studies, some researchers have provided more detailed molecular information about the function regions or epitopes of EDIII. For example, in Deng’s study [81], he found that the motif ^394^HHWH^397^, which was located within the terminai end of a β-pleated sheet of a JEV EDIII protein, was the minimal unit of linear epitope that was recognized by mAb 2B4. Importantly, this motif was highly conserved among JEV strains and also exists in WNV. This epitope can be recognized not only by JEV-positive swine serum, but also by WNV-positive swine serum. Mathengtheng and colleagues [1,82] applied serological assays using native and recombinant EDIII proteins as antigens to evaluate the detection and differentiation of tick- and mosquito-borne flaviviruses in the Free State providence, which demonstrated that the EDIII protein of flaviviruses has type-specific epitopes. Cecile’s study [83] showed that as a viral antigen, the *flavivirus* EDIII protein specifically captured the antibodies directed against WNV, JEV, or TBEV in spite of the well-known antigenic cross-reactivity between these flaviviruses. *Flavivirus* EDIII shows a similar function in antiviral studies, but its structure varies among strains. For example, the structure of YFV EDIII is arranged into three β-sheets containing nine β-strands (A, B, C, D, E, F, G, Cx, and Dx), which differs from that of other *flavivirus* structures. In YFV, the BC loop has one less amino acid than mosquito-borne and non-vector-borne viruses, but it is the same length as most tick-borne viruses. The special epitopes are associated with neutralizing YFV, DENV, WNV, and JEV, but are not consistently located in EDIII, such as DENV residues 284 and 305 (F-G loop); JEV residues 302, 306, 331, 332, and 333 (B-C loop) [49]; YFV residues 305 and 325 (B-C loop); and WNV residues 310 and 332 (B-C loop) [31]. The properties suggest that *flavivirus* structures are variable.

## 4. Envelope Proteins Applications

In most flaviviruses, as the major virion component, the multifunctional glycosylated E protein mediates infection to susceptible host cells, promoting entry by membrane fusion [84,85] and stimulating the production of neutralizing antibodies [50]. Thus, it is a potential candidate for *flavivirus* prevention and treatment. Notably, the E protein EDIII, which is thought to contain cell receptor-binding sites, mediates *flavivirus* infection in several ways [23]. To date, the E protein foci overlap in both vaccine and therapeutic target. It is used in vaccines and therapeutic applications as well as in viral detection because of its antigenicity [46,86]. Deng’s study [81] found the EDIII-specific linear epitope, ^394^HHWH^397^ of EDIII, was specifically identified by mAb 2B4, suggesting EDIII may be a potential diagnostic and therapeutic target. In Cecile’s study [83], as the viral antigen, the *flavivirus* EDIII protein specifically captured the antibodies directed against WNV, JEV, or TBEV in spite of the well-known antigenic cross-reactivity between these flaviviruses, which stimulated EDIII to be used as an antigen for the serological diagnosis of *flavivirus* infections. The *flavivirus* E protein has many potential applications (Table 1).

## 5. Discussion

Viruses enter susceptible cells by receptor-mediated endocytosis, and flaviviruses enter the cytoplasm by viral glycoprotein-mediated membrane fusion at a low pH [37,100]. All viral fusion proteins, including the E protein, have two membrane-interacting elements: A C-terminal transmembrane anchor that supports the proteins in the viral membrane and a hydrophobic region (fusion peptides or fusion loops) that interacts with the cell membrane. In the active fusion state, these elements change from dimers to trimers [44]. Fusion proteins such as E can reduce the high kinetic barrier from lipid-bilayer fusion by a battery of membrane-related conformational rearrangements [101]. Investigators are interested in using E proteins for diagnostic purposes and vaccine candidates. The E protein is a major antigenic target in neutralizing antibody recognition by blocking viral attachment, membrane fusion, and endocytosis [39,102]. Moreover, a large number of neutralizing antibodies recognize epitopes located on domain III, suggesting the EDIII protein may be a useful tool in the detection and differentiation of flaviviruses [1]. Recent studies have highlighted a new class of epitopes in Dengue virus that are present only in the dimeric form of the envelope glycoprotein [103,104,105]. Selective pressure from the host immune system can propel viral gene evolution, particularly that of the E gene; hence, genetic changes can render viruses resistant to anti-E neutralizing antibodies [39]. The E protein is associated with low-pH-dependent membrane fusion between viruses and host cells [106]. The three separate structural domains execute numerous but associated functions in *flavivirus* infection. EDII and EDIII of the E protein synergize during interactions with cellular receptors. The differences in biophysical properties among the three domains of the E protein may correlate with the variable *flavivirus* tolerance to environmental conditions [107]. The changes in *flavivirus* E protein structure may significantly affect viruses and ligand interactions, such as in cell receptors, drugs, and antibodies. Because of the conservatism of E proteins among flaviviruses and the intimate connection between DENV and ZIKV, Dejnirattisai [108] used the E protein of DENV to detect the infection of ZIKV.

In future studies, it is imperative to either design inhibitors that compete with the E protein to interact with cell receptors or medicines that directly interact with the E protein. It is difficult to ascertain the factors that affect viral entry, so a profound understanding and in-depth analysis of E protein structure and function will be a breakthrough in *flavivirus* research and will also help us to sufficiently understand *flavivirus* biological properties and virus-cell interaction mechanisms. Although many biological *flavivirus* properties have been reported, no efficient clinical drugs are available. More fundamental studies on E proteins in *flavivirus* infections should be conducted in the future.

## Figures and Tables

**Table 1:** E protein applications.

Structures	Viruses	Strains	Gen Bank Accession Numbers	Application Types	References
EDIII	YFV	17D strain	JX949181.1	vaccine	[87]
	JEV			vaccine	[88]
	TBEV			vaccine	[89]
	TMUV	FX2010		ELISA	[85]
	WNV			therapeutic	[78,90]
E and EDIII		NY99-382	AF196835	vaccine	[91]
E				mAb	[92]
		New York 1999 strain	FJ151394	diagnostic reagent	[93]
EDIII	DENV	B5/ H241	AF289029/U18433	neutralizing epitopes	[94]
		Hawaii/New Guinea-C/Guanxi-80-2/H241		vaccine	[95]
EDI/EDII hinge		rDENV-4	1683917	vaccine	[61]
E		DENV-1 WestPac74		mAb	[96]
		DENV-2 S-16803			
		DENV-3 CH-53489			
		DENV-4 TVP-376			
		ZIKV H/PF/2013			
		ZIKV PRVABC59			
	ZIKV	H/PF/2013	KJ776791.2	peptide drugs	[69]
		50 strains		peptide vaccine	[97,98]
		5IRE		diagnostic sites	[99]

E: Envelope, EDI/II/III: Envelope Domain I/II/III, YFV: Yellow Fever Virus, JEV: Japanese Encephalitis Virus, TBEV: Tick Borne Encephalitis Virus, TMUV: Tembusu Virus, DENV: Dengue Virus, ZIKV: Zika Virus, mAb: Monoclonal antibody, ELISA: Enzyme-linked immunosorbent assay.
